# A review of novel optical imaging strategies of the stroke pathology and stem cell therapy in stroke

**DOI:** 10.3389/fncel.2014.00226

**Published:** 2014-08-14

**Authors:** Markus Aswendt, Joanna Adamczak, Annette Tennstaedt

**Affiliations:** In-vivo-NMR Laboratory, Max Planck Institute for Neurological Research, KölnGermany

**Keywords:** optical neuroimaging, non-invasive, stem cell therapy, stroke, bioluminescence imaging, fluorescence imaging

## Abstract

Transplanted stem cells can induce and enhance functional recovery in experimental stroke. Invasive analysis has been extensively used to provide detailed cellular and molecular characterization of the stroke pathology and engrafted stem cells. But post mortem analysis is not appropriate to reveal the time scale of the dynamic interplay between the cell graft, the ischemic lesion and the endogenous repair mechanisms. This review describes non-invasive imaging techniques which have been developed to provide complementary *in vivo* information. Recent advances were made in analyzing simultaneously different aspects of the cell graft (e.g., number of cells, viability state, and cell fate), the ischemic lesion (e.g., blood–brain-barrier consistency, hypoxic, and necrotic areas) and the neuronal and vascular network. We focus on optical methods, which permit simple animal preparation, repetitive experimental conditions, relatively medium-cost instrumentation and are performed under mild anesthesia, thus nearly under physiological conditions. A selection of recent examples of optical intrinsic imaging, fluorescence imaging and bioluminescence imaging to characterize the stroke pathology and engrafted stem cells are discussed. Special attention is paid to novel optimal reporter genes/probes for genetic labeling and tracking of stem cells and appropriate transgenic animal models. Requirements, advantages and limitations of these imaging platforms are critically discussed and placed into the context of other non-invasive techniques, e.g., magnetic resonance imaging and positron emission tomography, which can be joined with optical imaging in multimodal approaches.

## INTRODUCTION

The stroke pathology and regeneration processes induced by endogenous mechanisms or engrafted stem cells have been studied extensively. Invasive studies – including immunohistochemistry, autoradiography, electrophysiology, and molecular biology – revealed the ischemic cascade of pathological and protective signaling events (Zhang and Chopp, 2009; Iadecola and Anrather, 2011). New neurons are found in the rat striatum after experimental stroke (Arvidsson et al., 2002), but neurogenesis and functional neuronal integration seem alone not to be able to restore brain function. In this line, exogenous stem cells, e.g., neural stem cells (NSCs) derived from embryonic or induced-pluripotent stem cells, have been implanted in experimental rodent models of stroke and found to increase functional recovery in many studies (Bliss et al., 2007; Oki et al., 2012). However, the interplay of stem cells with the injured host tissue and the mode of action of engrafted cells in the longitudinal time profile of stroke regeneration have to be deciphered before clinical translation.

**FIGURE 1:**
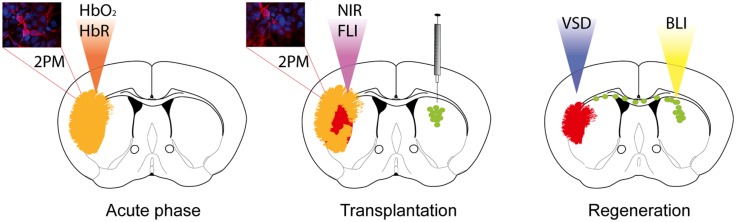
**Optical neuroimaging strategies to target the stroke pathology and regeneration processes upon stem cell grafting.** Two-photon microscopy (2PM) can be used to monitor blood flow changes during the acute phase, as well as changes in the cortical neuronal network over time. Optical intrinsic imaging utilizes the different absorption behavior of oxygenated (HbO_2_) and deoxygenated hemoglobin (HbR) to resolve changes in blood flow. Near-infrared probes have been designed to target, e.g., apoptotic cells or immune cells specifically by fluorescence imaging (FLI). Functional reorganization can be monitored by application of voltage-sensitive dye (VSD) imaging. Transplanted stem cells expressing bioluminescence and fluorescence imaging reporters allow longitudinal monitoring by bioluminescence imaging (BLI) and histological validation.

We review here optical imaging as one promising approach to shed new light on structural and functional components of stem cell therapy in stroke. We introduced fluorescence and bioluminescence imaging (FLI and BLI) which have been extensively developed in the last decade to meet the criteria of a highly sensitive and minimally invasive set-up (**Figure 1**). We provide a selection of recent publications related to stroke and/or stem cell transplantation in rodents, which we find appropriate to introduce current possibilities and constraints of optical neuroimaging. In addition, selected references – in line with most preclinical optical imaging studies – refer to mice or rats and exclude, e.g., stroke studies in non-human primates (Bihel et al., 2010) or the zebrafish (Walcott and Peterson, 2014).

### OPTICAL NEUROIMAGING

**FIGURE 2:**
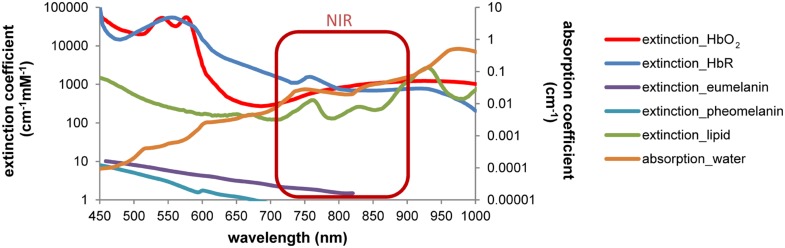
**Wavelength-dependent absorption in tissue restricts the optical imaging spectrum.** Light absorption by hemoglobin, melanin, lipids and water is wavelength-dependent. Differences can be used to discriminate, e.g., oxy- and deoxyhemoglobin by spectral analysis. A window of low absorption exists in the near-infrared range (∼700–900 nm). Absorption and extinction coefficients from Palmer and Williams (1974), Cheong et al. (1990), Pope and Fry (1997), Prahl (1999) and Sarna and Swartz (2006), displayed in log scale.

Molecular imaging aims to visualize cellular and molecular events – related to physiological or pathophysiological processes – in the living subject, e.g., by genetically linked imaging reporters (Massoud et al., 2008a). Based on the first non-invasive experiments with superficial sources, optical neuroimaging has been so far most effectively implemented for brain tumor studies (Massoud et al., 2008b) and less for neurological disease models or endogenous/exogenous NSCs in which sensitivity is essential. Compared to superficial sources, the brain appears to be a very difficult organ to be penetrated and explored by light. The natural multilayer barrier of blood, meninges, bone and skin covers all neural cells. Despite the extensive technical developments in optical imaging, major challenges of light absorption and scattering, autofluorescence, low spectral resolution and quantification still need to be considered (Shah and Weissleder, 2005; Hillman, 2007; Sutton et al., 2008). Among these physical limitations, light absorption and scattering are the main cause that affects *in vivo* optical approaches. Absorption is mainly driven by pigments/chromophores (hemoglobin and bilirubin in the blood, myoglobin in the muscles, pheo- and eumelanin in the skin) and also by water and lipids (**Figure 2**). Brain tissue requires continuous blood supply, which implies strong light attenuation by absorption. Efficient light propagation through the brain is provided in a naturally existing window of low absorption in the near-infrared (NIR, ∼700–900 nm; Frangioni, 2003). Despite absorption effects, light is scattered at inter- and intracellular membrane boundaries due to differences in the refractive index *n* (ratio of the speed of light in vacuum and speed of light in the material), e.g., extracellular fluids (*n*= 1.335) and triglycerides (*n*= 1.491; Ross, 1967). The brain parenchyma is composed of many of these specific boundaries, most prominently white and gray matter, leading to light scatter. Improving the imaging set-up, e.g., by advanced technical devices or imaging reporters with higher sensitivity will certainly facilitate imaging in small animals. But it should be noted that the physical factors leading to light attenuation and scattering and the insertion of an imaging transgene definitely limit the application on small animals and preclude optical neuroimaging in humans. As this review is focused on recent optical imaging applications but not on the physical principles, we refer the interested reader to a technical review of Schulz and Semmler (2008) and the comprehensive book Molecular imaging: Principles and Practice edited by Weissleder and Gambhir (2010). The following two chapters introduce FLI and BLI techniques with a focus on optical neuroimaging, describe useful imaging reporters and recent studies. Finally, we discuss the importance of cell-specific imaging and the benefit of combining different imaging techniques.

## FLUORESCENCE IMAGING

*In vivo* FLI uses a set-up similar to fluorescence microscopy consisting of a light source, fluorescence filters and a sensitive charge-coupled device (CCD) camera. But in addition, the set-up is housed in a light-tight chamber to collect fluorescence emission exclusively from the anesthetized animal at the macroscopic level (Rao et al., 2007). To penetrate the mouse skull efficiently, excitation with NIR laser light either through the mouse head [transillumination fluorescence imaging (TFI)] or from top [fluorescence reflectance imaging (FRI)] is applied (Frangioni, 2003; Klohs et al., 2006). New systems can perform transillumination while the mouse is rotated through 360° to allow photon acquisition from multiple projections [fluorescence tomography (FMT); Deliolanis and Ntziachristos, 2013]. Nevertheless, some applications still require exposure of the skull and removal of the skin. In general, only NIR fluorescence is efficient enough for *in vivo* neuroimaging due to the strong attenuation of shorter wavelengths (<700 nm). FLI has been adapted to the mouse brain application in the micro- and macroscale to visualize stem cells or stroke-related functional changes based on fluorescent proteins (FPs), fluorescent dyes, and endogenous chromophores.

### FLUORESCENCE PROTEINS

The discovery of the green FP (GFP) from the jellyfish *Aequorea victoria* paved the way for a universal marker for cell structures and cellular processes detectable by fluorescence microscopy (Chalfie et al., 1994). The diversity of FPs has increased since then tremendously by mutating the original GFP sequence and cloning FP from distant species like crustaceans. Such FPs can be expressed in mammalian cells, including stem cells and transgenic mice without signs of toxicity (Shaner et al., 2005). Smart multi-label approaches like the brainbow toolbox have been developed to mark neurons with many different FPs (Cai et al., 2013). However, *in vivo* FLI is challenged by a variety of factors: the excitation/emission wavelength, the brightness being determined by the quantum yield (QY, the ratio of photons emitted to photons absorbed during excitation), the extinction coefficient (EC, determining how strongly light is absorbed), the FP maturation rate, photostability, pH stability, and aggregation potential (Chudakov et al., 2010). FPs for *in vivo* neuroimaging will profit from novel – much more efficient – NIR probes (Shcherbakova and Verkhusha, 2013).

### FLUORESCENT DYES

**FIGURE 3:**
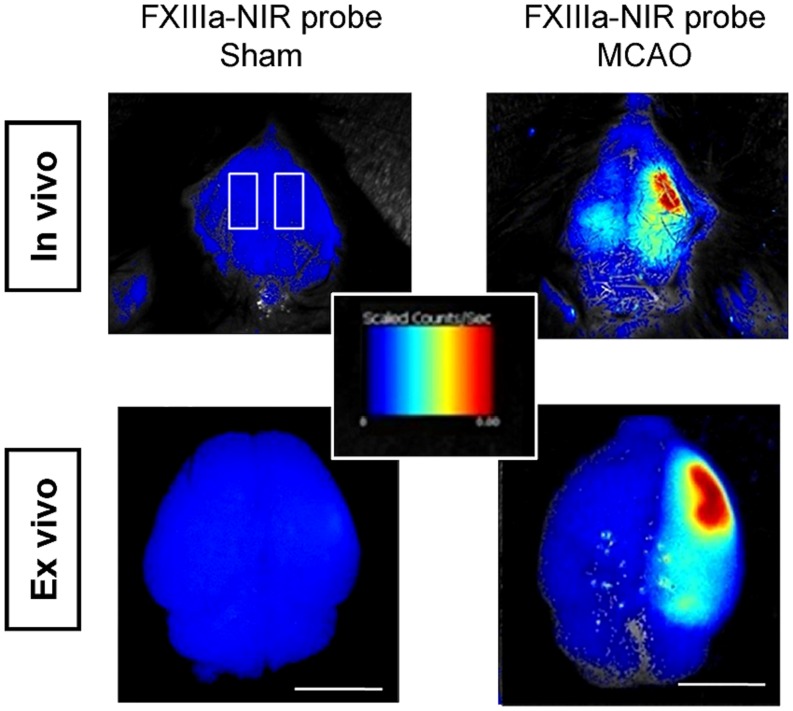
***in vivo* FLI of thromboembolic-stroke.** Fibrin deposition is visualized with a near-infrared probe against the activated coagulation factor XIII (FXIIIa; A15) in a thromboembolic model of stroke. Scale bar 5 mm. Images adapted with permission from Zhang et al. (2012).

Several chemical probes have proven long term labeling of stem cells, including chloromethylfluorescein diacetate (CMFDA or CellTracker) and long chain carbocyanine dyes (like DiI, DiO, DiD, and CM-DiI; Sutton et al., 2008; Mäkinen et al., 2013; Boehm-Sturm et al., 2014). CMFDA and DiD labels were found to be stable for up to 4 weeks in human ES-cell derived neural cells *in vitro* (Mäkinen et al., 2013). Stem cell labeling prior to implantation is efficient but cell tracking is restricted to *ex vivo* fluorescence microscopy (Jablonska et al., 2010; Boehm-Sturm et al., 2014). *In vivo* data has been acquired with NIR cyanine dyes (Cy) – especially Cy5.5 (excitation 675 nm, emission 694 nm). Targeted probes have been designed to visualize key components of the stroke pathology. Inflammatory processes are monitored by an fluorescence-labeled antibody against the inflammatory receptor CD40 expressed on immune cells (Klohs et al., 2008) and dead cells can be targeted by the cell death marker Annexin A5 (Bahmani et al., 2011). In addition, Zhang et al. (2012) investigated an optical method to detect fibrin deposition, which leads to thrombosis – responsible for 80% of human stroke. In a mouse model of thromboembolic stroke, a NIR probe was injected, which is recognized by the activated coagulation factor XIII (FXIIIa), an important mediator of thrombosis or fibrinolytic resistance (**Figure 3**). Numerous probes have the potential to visualize stroke-related pathologies and host reactions, e.g., blood–brain-barrier (BBB) breakdown, infiltrating immune cells and (de-) myelination (Wang et al., 2011; Eaton et al., 2013) but have not been tested in animal models of stroke yet. NIR probes have been successfully used to simultaneously image cell death and BBB-disruption in traumatic brain injury (Smith et al., 2012). Smart probes have been designed to report activity of matrix metalloproteinases, which are highly upregulated after stroke (Klohs et al., 2009). In order to overcome the low sensitivity of FPs to label stem cells before transplantation, the new class of 2–8 nm small fluorescent quantum dots (QDs) holds great promise to overcome the low sensitivity of FPs to label cells. QDs provide surpassing absorbance, high QY, narrow emission bands and high resistance to photobleaching (Frangioni, 2003; Michalet et al., 2005). Sugiyama et al. (2011) could show that bone marrow stromal cells, labeled with QDs for the NIR, can be detected non-invasively up to 8 weeks after transplantation in the rat brain.

### FLUORESCENCE NEUROIMAGING AT THE MICRO- AND MACROSCALE

Two-photon microscopy (2PM) is the method of choice to obtain detailed structural information of neural tissue *in vivo* [also referred to 2P laser scanning microscopy (2PLSM)]. A pulsed infrared laser is used to excite fluorophores by the combined power of two long-wavelength photons (Sigler and Murphy, 2010), which promotes better sample penetration, higher resolution, less light scatter, and less photo-damage compared to *in vivo* confocal microscopy (Belluscio, 2005). 2PM has also been applied to freely moving animals equipped with a fiber-based endoscopic system (Helmchen et al., 2001). Using 2PM implies the limitation to the first cortical layers within the mouse brain, imaging subcortical structures requires a cranial window (Dombeck et al., 2010; Shih et al., 2012). Fluorophores are essential for 2PM, either by injectable tracers, like intravenous bolus injection of fluorescein-conjugated dextran to target blood vessels and blood flow (Shih et al., 2012), or FPs expressed by specific cell types (Belluscio, 2005). Label-free 2PM of the living mouse brain has been reported by Witte et al. (2011) by using intrinsic non-linear light interactions, referred to second- and third-harmonic generation, which can be applied to visualize, e.g., myelin (Farrar et al., 2011). Zhang and Murphy (2007) and Sigler and Murphy (2010) could identify with 2PM to which extent reduced blood flow in stroke leads to changes in synaptic circuitry. Recently, three-photon microscopy (3PM) extended the depth resolution to 1 mm by using longer wavelengths (1,700 nm, equivalent to a one-photon excitation of ∼560 nm), which are less attenuated by tissue and appropriate to excite a variety of existing fluorophores (Horton et al., 2013).

A common way to measure neuronal activity indirectly *in vivo* is functional magnetic resonance imaging (MRI) on the basis of the hemodynamic response, thus the locally dynamic changes in oxy- and deoxyhemoglobin (HbO_2_ and HbR). Differences in absorption of HbO_2_ and HbR can be used by intrinsic imaging to record cortical activity on a sub-second time scale based on the changes in blood oxygenation (Ts’o et al., 1990). The exposed cortex is illuminated sequentially by light of different wavelengths (multi-spectral reflectance imaging). Images are recorded which are particularly sensitive to changes in HbO_2_ and HbR concentration. At wavelengths where HbO_2_ and HbR absorption is the same (isosbestic points), changes in total hemoglobin concentration can be measured (Hillman, 2007). Abookasis et al. (2009) used NIR illumination in ischemic rat barrel cortex to generate maps of light absorption, scattering properties and tissue hemoglobin concentration. Dynamic changes in cerebral blood flow are acquired by laser speckle-flow imaging, which is using a similar set-up to intrinsic imaging but with a laser diode as light source. The laser speckle pattern is caused by the coherent laser light scattering within the brain, which is dependent on the movement of red blood cells over time (Hillman, 2007). A cranial imaging window is necessary for repeated light illumination of the exposed cortex (Armitage et al., 2010). Both systems can be combined for simultaneous blood oxygenation and flow imaging during stroke in rats (Steimers et al., 2011). However, blood flow dynamics and neuronal structures are still best resolved with 2PLSM (Shih et al., 2012).

**FIGURE 4:**
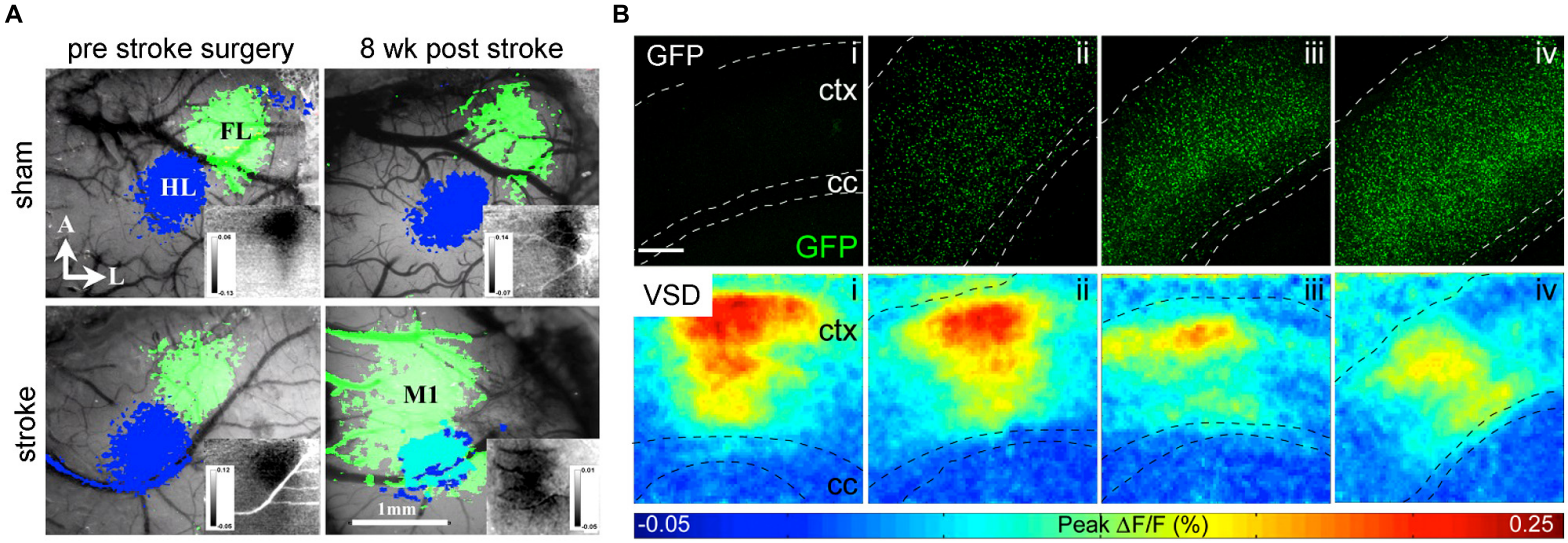
**VSD imaging detects macroscopic changes in functional networks after stroke and implantation of NSCs.** **(A)** Changes of the functional representation of the forelimb (FL) cortex area toward the peri-infarct primary motor cortex M1 and hindlimb (HL) area appear 8 weeks upon photothrombotic stroke but not sham surgery. Reprinted by permission from MacMillan Publisher Ltd: Molecular Therapy (Brown et al., 2009). **(B)** With increasing number of cortical grafts (confocal images upper row), the amplitude of cortical activation is reduced as displayed in color-coded maps (lower row) of cortical activation depicting the maximum of mean fluorescence intensity change (F/F_0_) within 1024 ms recording interval. Images adapted with permission from Weerakkody et al. (2013).

Grinvald and colleagues paved the way for a direct method to optically record neuronal activity: voltage sensitive-dyes (VSDs; Orbach et al., 1985) and voltage sensitive-proteins (VSFPs; Sakai et al., 2001). Both, VSDs and VSFPs respond to changes in transmembrane voltage in the millisecond time scale, by changing their fluorescence properties [but resolving single action potentials is still limited (Akemann et al., 2009)]. Several studies have recently applied VSDs on experimental models of stroke to image functional reorganization of forelimb cortex areas (Brown et al., 2009; **Figure 4A**) and long-lasting impairments on the processing of sensory stimuli by the forelimb somatosensory cortex (Sweetnam and Brown, 2013). Weerakkody et al. (2013) applied VSD imaging to study the cortical activity after implantation of NSCs into the ventricle of naïve mice. They found that high-density engraftment of non-integrating NSCs leads to functional defects in cortical layers (Weerakkody et al., 2013; **Figure 4B**).

## BIOLUMINESCENCE IMAGING

**FIGURE 5:**
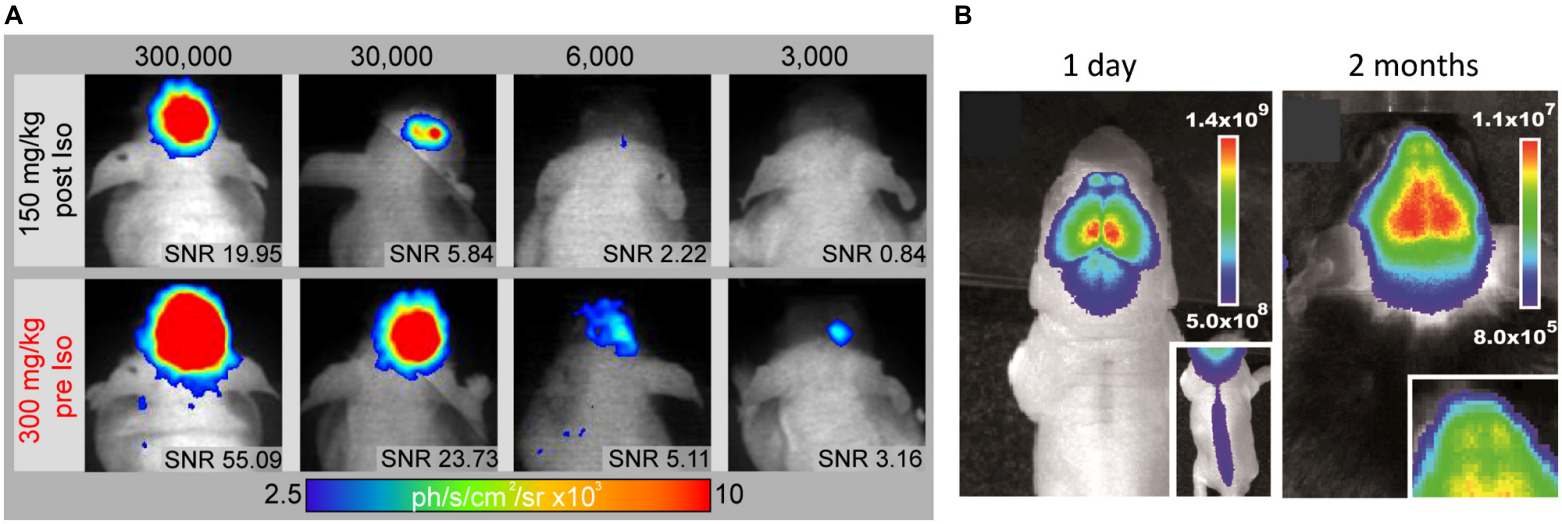
***In vivo* monitoring of endogenous and implanted stem cells.** **(A)** The improved BLI protocol (300 mg/kg Luciferin pre Isofluran anaesthesia) promotes substantial higher photon emission compared to standard protocol (150 mg/kg Luciferin post anesthesia) and the lowers the detection limit to 3,000 engrafted NSCs *in vivo*. Signal-to-noise ratio (SNR) above 3 was defined as a limit for reliable cell graft detection. **(B)** Bioluminescence images of a 1 day and 2 month old doublecortin-luciferase mouse for *in vivo* imaging of endogenous neurogenesis (Insert shows neurogenesis in the spinal cord and the olfactory bulbs, respectively). Images modified with permission from Aswendt et al. (2013) and Couillard-Despres et al. (2008).

*In vivo* BLI uses, similar to fluorescence imaging (FLI), a CCD camera housed in a light-tight chamber to collect photon emission from the anesthetized animal. But instead of an excitation source, photons are emitted when the intracellular enzyme luciferase oxidizes its substrate luciferin. As luciferase is only expressed in transgenic mammalian cells, there is only negligible BLI background signal and emitted photons can be detected with surpassing sensitivity through the intact skull (Keyaerts et al., 2012a) – even from freely moving awake animals (Keyaerts et al., 2012b). BLI is a high throughput technique, scalable from *in vitro* to *in vivo*, highly non-invasive and ease of use. Similar to FLI, the spatial resolution is limited to several mm (Massoud and Gambhir, 2003). Although the number of photons emitted is proportional to the number of luciferase molecules (De Wet et al., 1987), quantification of *in vivo* BLI is also challenged by biological factors (e.g., substrate bio-distribution, luciferase expression, stability, and inhibition) and physical factors (e.g., emission wavelength, light attenuation by overlying tissue; Keyaerts et al., 2012a). The percentage of transmitted light is linearly decreasing with depth of the bioluminescent source in the rodent brain (Pesnel et al., 2011). However, this could be used to predict the extent of optical attenuation for correct quantification (Virostko and Jansen, 2009). BLI quantification *in vitro* relies on excess conditions of ATP and oxygen. But *in vivo* only 5% of the systemic administered luciferin reaches the brain (Berger et al., 2008), as it has to pass several biological barriers to reach the cell of interest and distribution is dependent on the hemodynamic rate (Keyaerts et al., 2012a). Luciferin can pass freely through the BBB, but eﬄux transporters like ABCG2 actively pump it back to the lumen (Bakhsheshian et al., 2013) and BBB disruption during stroke may affect biodistribution. Recently, luciferin derivates have been developed to boost sensitivity especially for the mouse brain even at lower doses (Evans et al., 2014). In addition, we proposed an optimized neuroimaging protocol, which minimizes inhibitory effects on the luciferase-luciferin reaction while maximizing the photon detection by a factor of two over conventional experimental protocols and provides detection of 3,000 NSCs *in vivo* (Aswendt et al., 2013; **Figure 5A**).

### LUCIFERASES FOR *IN VIVO* IMAGING

**FIGURE 6:**
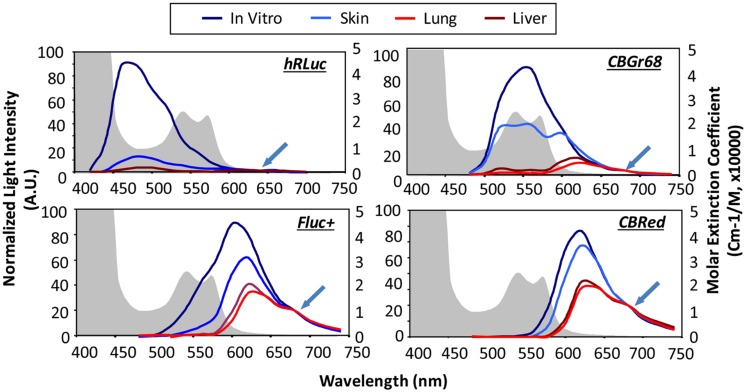
***In vivo* emission spectra of bioluminescent reporters.** Emission spectra from luciferase expression in skin, lung, and liver are compared with those from labeled cells in culture showing tissue-induced light attenuation in the blue spectrum. Photon fluxes are normalized to the values at 680 nm for beetle luciferases and 640 nm for Renilla luciferase (indicated at green arrow), where absorption is minimal. Hemoglobin absorption curves are plotted as background (shaded in gray). hRLuc, Renilla reniformis; CBGr68, click beetle green; CBRed, click beetle red; Fluc+, Firefly luciferase. Reproduced with permission from Zhao et al. (2005).

Luciferase enzymes occur naturally in numerous luminous species, such as the North American firefly [firefly luciferase (Fluc)], click beetles [click beetle luciferase (CBR)], the sea pansy *Renilla reniformis* [Renilla luciferase (Rluc)], and the copepod *Gaussia princeps* [Gaussia luciferase (Gluc); Mezzanotte et al., 2013]. There are species-specific changes in the substrate, co-factors and emitted wavelength. Gluc and Rluc emit in the blue spectrum and therefore are not optimal for *in vivo* use, as it gets strongly absorbed when traveling through tissue (see **Figure 1**). Fluc and CBR, on the other hand, have a strong component above 600 nm (**Figure 6**) resulting in less absorption (Zhao et al., 2005). Changes in one single amino acid of the luciferase can already result in wavelength shifts. Extensive mutagenesis was performed to create red and green shifts and improve pH-tolerance and thermostability (Miloud et al., 2011; Jathoul et al., 2012). To answer the question, which luciferase fits best the brain application, we recently evaluated the codon-optimized Luc2, the red codon-optimized mutant PpyRE9 (Liang et al., 2012), the codon-optimized hRluc and the green CBG99. BLI signal from transduced cells *in vivo* after intracerebral transplantation showed photon emission decrease in the order of Luc2, CBG99, PpyRE9 to hRluc (Mezzanotte et al., 2013). Most importantly, as the green part of emission spectra gets strongly absorbed, luciferases with a high QY and red-shifted emission are preferable. Multicolor BLI, e.g., to distinguish two cell types becomes possible by combining Luc2/hRluc [through the not cross-reacting substrates luciferin and coelenterazine (Bhaumik and Gambhir, 2002)] or CBG99/PpyRE9 [through spectral unmixing of the green and red emission light (Mezzanotte et al., 2013)].

### BIOLUMINE SCENE IMAGING OF ENDOGENOUS AND EXOGENOUS STEM CELLS FOR STROKE REPAIR

Stem cell therapy has already been proven beneficial for stroke recovery (Bliss et al., 2007; Oki et al., 2012), however, the mechanisms of action needs further investigation. BLI provides unique information about the dynamics of NSCs, such as location, migration and proliferation. Exogenous stem cells have to be transgenically modified to express a luciferase protein in order to allow longitudinal *in vivo* optical imaging after implantation. By using bicistronic vectors for expression of two transgenes, e.g., a bioluminescent reporter for *in vivo* BLI together with a fluorescent reporter (e.g., EGFP) provides the opportunity to also detect engrafted cells by invasive methods such as histology, microscopy, RT-PCR and Western blot. Notably, some luciferases, as Fluc, are dependent on ATP, thus serving as a non-invasive viability marker of engrafted stem cells to assess survival *in vivo*. Using this biochemical relationship Boehm-Sturm et al. (2014) investigated whether the peri-infarct region is a location permissive for stem cell survival. Their multimodal approach of 19F-MRI and BLI revealed that human NSCs after intracerebral implantation into the peri-infarct region show same survival behavior as when implanted into healthy brain tissue. *In vivo* BLI has also been used to assess the recruitment of NSCs in stroke mice after contralateral parenchymal, intra-ventricular (Kim et al., 2004) or intra-arterial injection (Rosenblum et al., 2012). Murin neural precursor transfected to express Fluc showed strong migrational activity toward the lesioned hemisphere when transplanted contralaterally. Despite the rather poor spectral resolution of BLI compared to other imaging modalities like MRI, Kim et al. (2004) could detect the midline crossing as early as 7 days after grafting (Quattromani et al., 2014). Also intraventricular injection of this cell line into stroke mice resulted in strong recruitment to the lesioned area (Quattromani et al., 2014). Intra-arterially injected murine neural progenitors transduced to express Gluc were found to reach the brain, with largest recruitment to the brain when injected 3 days after hypoxia-ischemia (Cordeau et al., 2008).

The second source of stem cells for stroke repair is the pool of endogenous stem cells, e.g., within the subventricular zone. These cells can be either targeted by *in vivo* transduction with a viral vector to express Fluc (Reumers et al., 2008), or by designing a neural progenitor-specific reporter mouse, in which Fluc expression is controlled by the doublecortin (DCX) promotor (Couillard-Despres et al., 2008). Both approaches allow quantitative observation of adult neurogenesis (**Figure 5B**) and represent useful non-invasive tools to investigate the therapeutic potential of endogenous stem cells for stroke recovery. Using conditional viral vectors, Vandeputte et al. (2014) labeled Nestin-positive cells of the subventricular zone prior to induction of photothrombotic stroke and followed the recruitment to the lesion for 90 days. Photothrombotic stroke is a variation of ischemic stroke models, which produces localized cortical strokes based on microvascular thrombosis after localized photosensitive dye activation. Already 2 days after stroke, the number of endogenous neural progenitor cells increased and translocated to the lesion site, peaking at 14 days and declining thereafter (Vandeputte et al., 2014). The time profile of the endogenous neural progenitor cell proliferation in response to ischemic stroke of the middle cerebral artery territory was investigated using the DCX reporter mouse model (Adamczak, 2013). A similar early rise in proliferation was observed within the first week. Other aspects of neural replacement have been investigated by non-invasive BLI. For example neural responses to stroke by Gravel et al. (2011) using a multimodal transgenic mouse targeting the growth associated protein GAP-43. BLI could show that the nervous tissue-specific GAP-43 is silent in adult neurons, but up-regulated after neural injury and contributes to neurite outgrowth as part of the regeneration process after stroke. Post-stroke neurogenesis is effected by the inflammatory response (Kokaia et al., 2012), which can be monitored with a transgenic mouse model expressing Fluc under the control of the toll-like receptor (TLR) two promotor (Lalancette-Hébert et al., 2009). TLRs are expressed by cells of the innate immune system to identify damage-associated patterns released during cell damage. Quattromani et al. (2014) successfully used this mouse model to visualize that the inflammatory response in stroke mice is decreased when the animals had access to enriched environment in their cages. The inflammation post stroke was also found to be sex dependent using a transgenic mouse model with Fluc restricted to GFAP positive cells, predominantly astrocytes. While male mice showed a correlation of astrogliosis and infarct volume, such a correlation was missing in female mice (Cordeau et al., 2008).

## FUTURE DIRECTIONS

### CELL FATE IMAGING

Besides the possibility to track the viability of transplanted NSCs by BLI, luciferases have been linked to neural cell specific promoters to monitor differentiation. Such system was successfully used to trace the *in vivo* activation of neuronal differentiation by coupling Fluc to the NeuroD promoter which is active in neuronal precursor cells (Oh et al., 2013). Weak cell specific promoters can be enhanced by coupling them to a two-step transcriptional amplification (TSTA; Hwang do et al., 2008). An efficient *in vitro* method to increase reliability of BLI quantification was described as dual-reporter systems, which uses a constitutive promoter to image the localization, viability and quantity of transplanted cells and a cell specific promoter to monitor the differentiation degree in real-time based on two different luciferases (Kern et al., 2013; Xu et al., 2014). The dual-reporter system can be used to answer important questions, such as (i) the optimal type and number of NSCs to be administered, (ii) the route of administration, and (iii) the best time to administer cells after injury with higher accuracy compared to single reporter assays.

### COMBINATION OF NON-INVASIVE IMAGING TECHNIQUES

Optical imaging provides best sensitivity of 10^-15^–10^-17^ mol/l for visualization of stem cell therapy in stroke (Massoud and Gambhir, 2003). Among many recent technical developments, optoacoustic (photoacoustic) imaging holds great potential to visualize both, the stem cell graft location (Jokerst et al., 2012) and progression/reorganization of the stroke lesion (Kneipp et al., 2014) with one non-invasive imaging device. However, also the combination of optical imaging with other non-invasive imaging techniques like positron emission tomography (PET) and MRI is of interest, as complementary functional and structural information can be derived. A novel multimodal approach is the combination of PET and optical intrinsic imaging to characterize cortical spreading depression in ischemic rats (Gramer et al., 2014). PET imaging may also be useful to quantify the metabolic rate (via the glucose consumption) and oxygen distribution, two important factors in stroke pathology, and confounding factors for BLI. Another multimodal approach is the combination of MRI with BLI to characterize transplanted NSCs in the stroke pathology. Firefly bioluminescence was used as a viability marker while transplant location was visualized with iron oxide labeling by MRI for 8 weeks (Daadi et al., 2009). Sequential MRI/BLI was successfully employed to investigate the amount and distribution of intra-arterially (i.a.) versus intra-venously (i.v.) injected stem cells in an ischemia model, showing brain accumulation after i.a. but not after i.v. injection (Pendharkar et al., 2010). We have combined the advanced 19F MRI technique for unambiguous graft location on high-resolution structural MR images with BLI for monitoring cell viability (Boehm-Sturm et al., 2014). There, the murine NSCs were transplanted next to the stroke lesion in nude mice. But the graft location had no effect on the decline in cell survival over 14 days observation period. Further technical developments are needed to improve sensitivity, spatial resolution and integration of imaging techniques to facilitate co-registration of quantitative data (e.g., on database references as the Allen brain atlas, http://www.brain-map.org/). The combination of optical imaging with MRI data could be used to create 3D reconstructions to relocate cell function back to the anatomical structure, which is highly resolved by MRI (100 μm spatial resolution).

We believe that the application of the non-invasive imaging tools presented here will be essential to fully understand stroke regeneration and the potential of engrafted stem cells in pre-clinical trials – the prerequisite of an effective clinical therapy.

## AUTHOR CONTRIBUTIONS

Markus Aswendt, Joanna Adamczak and Annette Tennstaedt wrote the manuscript.

## Conflict of Interest Statement

The authors declare that the research was conducted in the absence of any commercial or financial relationships that could be construed as a potential conflict of interest.
